# Development and Path of Reclaimed Water Utilization Policy in China: Visual Analysis Based on CNKI and WOS

**DOI:** 10.3390/ijerph191911866

**Published:** 2022-09-20

**Authors:** Junjie Li, Xin Dai, Bei Zhang, Xuehang Sun, Bangfan Liu

**Affiliations:** 1 School of Public Administration, Yanshan University, Qinhuangdao 066004, China; 2 Hebei Public Policy Evaluation and Research Center, Qinhuangdao 066004, China; 3 Institute of Marxism, Chinese Academy of Social Sciences, Beijing 300712, China; 4 Institute of Marxism, Shandong University, Jinan 250100, China

**Keywords:** reclaimed water, policy, water resources, visual analysis

## Abstract

In this paper, CiteSpace and NVivo software were used for the knowledge graph visualization and content analysis of highly cited papers in the research literature on reclaimed water utilization policy in CNKI and WOS. The results showed the following: there was an upward trend in the number of papers on reclaimed water policy, papers in both databases attached great importance to research on this topic, and the research prospects for this topic are broad. The UK, Greece, Italy, the United States, and France have great influence in the field of reclaimed water utilization policy research. The international influence of China’s research on the topic needs to be improved. There is a lack of communication and cooperation among the subjects of reclaimed water utilization policy research, and a cooperative network with close and benign interactions has not yet been formed. The research hotspots of the topic in China are mainly focused on regional governance, with insufficient attention paid to policy and management, while foreign countries pay more attention to policy and management. Behavior guidance policy and black and smelly water will become research hotspots for domestic policies, while public perception, demand, drinking water, and carbon will become research hotspots for international policies. Domestic research on reclaimed water use policy in highly cited papers focused on water environment and ecological security management, while international research focused on the background of reclaimed water use policy and its implementation, with the main intention of optimizing the ascension path and making international research policies thematically stronger. The attitudes of domestic and foreign researchers regarding reclaimed water utilization policies are mainly rational and emotional, indicating that current policies have a degree of applicability. However, there are also obvious problems that will need to be addressed and improved, and there are substantial development prospects. In the future, research on reclaimed water utilization policies in China should strengthen top-level design, improve the policy system, and increase the supervision of policies to achieve optimization.

## 1. Introduction

Water resources are not only productive, but also represent a lifeline. To a certain extent, access to water resources determines the quality of human production and life. As the global ecological environment continues to attract more attention in all countries, the recycling of water resources is attracting more people’s attention. One of the basic aspects of water resource recycling is vigorously promoting the utilization of reclaimed water. Reclaimed water utilization is an important form of water resource recycling, and an inevitable choice for the ecologicalization and utilization of sewage. Solving the shortage of water resources and meeting the growing demand for water are of great significance [1]. The global distribution of water resources is not balanced. Different countries and regions have different amounts of water resources; some have large amounts, while others are seriously deficient. On the whole, China’s water resources are seriously deficient, especially in the three dimensions of per capita water resources, urban per capita water resources, and per capita water resources in northwest China, which are all at the global bottom level. The demand for water resources in China is huge. However, with the emergence of environmental problems caused by economic and social development, the problem of water pollution has not been eradicated, and the utilization of reclaimed water has not been systematically controlled. The proper use and distribution of reclaimed water are not only beneficial for alleviating the lack of water resources and unbalanced distribution, but they are also the only way to promote China’s green transformation and sustainable development [2]. Sorting out the evolution path and context of China’s reclaimed water utilization policies, examining the policy development trends, and putting forward targeted optimization countermeasures and suggestions are of great theoretical and practical significance.

The concept of reclaimed water has not been unified in theoretical research and practical work. In this paper, reclaimed water mainly refers to water that can be safely used after sewage and wastewater are properly treated to reach certain quality standards and meet certain use requirements. [3] The process of reclaiming water in this paper mainly refers to the reuse or recycling of sewage after treatment, and reclaimed water is equivalent to circulating water. Terms related to reclaimed water include middle water, upper water, lower water, sewage, wastewater, conventional water, unconventional water, common water, rainwater, seawater, and brackish water.

The terms middle water, upper water, and lower water were first used in Japan [4]. Upper water refers to drinking, tap, or clean water, and lower water includes domestic sewage, industrial wastewater, and other production and domestic drainage. Middle water refers to treated water with a quality between upper and lower water. Sewage is equivalent to lower water in the broad sense, and it refers to domestic sewage in the narrow sense. Sewage is referred to in this paper in the broad sense, that is, lower water. Rain, sea water, and brackish water are natural water resources, and refer to water in the sky, in the sea, and in points between seawater and freshwater, respectively. A lake canal is brackish water. In some research and practice, the sources of reclaimed water also include rainwater, seawater, and brackish water [5], but that is not the case in this paper; the main sources include domestic sewage and industrial wastewater. Conventional water refers to water that can be directly used in the traditional sense, that is, it meets certain quality standards and is convenient for direct use in daily production and life; it mainly includes surface water, groundwater, and tap water as well as softened water, steam water, and geothermal water that can be purchased in the market [6]. Common water refers to tap water, drinking water, and purified water used for human consumption. Unconventional water, which differs from conventional water in the traditional sense, mainly includes rainwater, reclaimed water, desalinated seawater, mine water, and brackish water; it can be used or recycled after treatment and can replace conventional water resources to a certain extent [7]. In this paper, reclaimed water has the same connotation as middle water, which belongs to the category of unconventional water.

A search for all articles with “recycled water” in the title was conducted in CNKI, and 2676 articles (as of 2022-8-28) were retrieved. Among them, the earliest studies were published in 2003, and the most were published in 2012 (199 articles). The authors with the most published articles were Honglu Liu and Wenyong Wu of the Beijing Institute of Water Resources, with 34 and 32 articles, respectively. Of these, the most cited articles were “Heavy metal pollution risk of reclaimed water irrigation on soil and crops in Beijing” (cited 127 times) [8] and “Experimental study on the influence of short-term reclaimed water irrigation on the distribution of heavy metals in soil-crop” (cited 65 times) [9]. Peiling Yang of the College of Water Resources and Civil Engineering at China Agricultural University and Yiping Gan of Beijing Urban Drainage Group Co., Ltd. published 31 articles. Among them, the most cited articles were “Experimental study on the influence of reclaimed water irrigation on soil physical and chemical properties” [10] (cited 107 times) [10] and “Application and research progress of biological filter process wastewater and reclaimed water treatment” (cited 40 times) [11]. In addition to the above articles, the 10 most frequently cited papers were “Current Situation and Utilization prospect of urban Reclaimed Water Reuse” [12], “Discussion on the Water Quality Requirements of Reclaimed Water Reuse for Landscape Water” [13], “Research on the Influence of Reclaimed water Irrigation on Soil Performance and Soil Microorganisms” [14], “Standard Comparison and Technical and Economic Analysis of Reclaimed Water Reuse” [3], “The experience analysis of reclaimed water utilization in California and its enlightenment to China” [15], “Discussion on the quality standard of reclaimed water used in landscape water” [16], “Problems and countermeasures of reclaimed water reuse in landscape water” [17], “Review of reclaimed water utilization in foreign countries” [18], “Health risk exposure assessment of reclaimed water use” [19], and “Research progress on the effect of reclaimed water recharge on groundwater quality” [20].

The subject words of the 2672 articles mainly included “reclaimed water” (1756 instances of the term), “reclaimed water irrigation” (328), “reclaimed water reuse” (259), “reclaimed water utilization” (258), “reclaimed water treatment” (83), “Beijing” (73), “urban reclaimed water” (71), “landscape water” (68), “groundwater” (51), “reclaimed water landscape” (44), “reclaimed water resources” (41), “reclaimed water drip irrigation” (36), “sewage treatment plant” (36), “heavy metals” (35), “Tianjin” (33), “advanced treatment” (26), “circulating cooling water” (23), “eutrophication” (22),”reclaimed water project” (22), and “treatment technology” (21). 

In addition, a search for articles with “recycled water” in the title since 2019 was conducted in CNKI. There were 472 articles in total, and the top 10 articles cited were: “Review of research status of reclaimed water reuse” [21], “Effects of different reclaimed water irrigation methods on bacterial community diversity and pathogen abundance in soil-pepper system” [22], “Root irrigation simulation and layout parameter prediction of reclaimed water Yongquan based on HydrUS-3D model” [23], “Effects of reclaimed water irrigation level on distribution of heavy metals and pathogenic bacteria in soil” [24], “Variation of COD_(Cr) degradation coefficient and its influencing factors in the reclaimed water replenishment river North Canal” [25], “Influence of different potassium fertilizers on Cd of soil-crop system under reclaimed water irrigation” [26], “Research on public cognition and acceptance intention of reclaimed water in Xi’an City” [27], “Comparison of purification ability of reclaimed water by floating plants” [28], “Research on the development strategy of reclaimed water utilization in Beijing” [29], and “Study on the efficiency and mechanism of ‘Pond + Wetland’ coupling system in purifying reclaimed water replenished low C/N river and lake water bodies” [30]. Among the 472 articles, the use of subject words was as follows: “reclaimed water” (313), “reclaimed water irrigation” (49), “reclaimed water utilization” (44), “reclaimed water reuse” (39), “reclaimed water drip irrigation” (12), “reclaimed water treatment” (9), “Beijing” (8), “landscape water body” (8), “groundwater” (8), “microbial community” (7), “thermal power plant” (6), “sewage plant” (6), “Old Summer Palace” (6), “research on influencing factors” (6), “domestic reclaimed water” (6), “reclaimed water stable mixture” (6), “advanced treatment” (6), “reclaimed water infiltration” (6), “reclaimed water and groundwater” (5), and “heavy metals” (5).

From the above analysis, it can be seen that research on reclaimed water in the CNKI literature is mainly focused on the treatment technology, technical standards, nature, distribution, classification, value application, and benefit analysis of reclaimed water, and there is a lack of rich, in-depth policy analysis regarding reclaimed water utilization. CNKI has collected more than 90% of journal and graduate papers and articles published in major newspapers in the past 40 years. Although in that time, especially in the past 20 years, many Chinese scholars have tended to publish their papers in WOS-listed journals, especially in English journals, the literature included in CNKI still reflects the research interests of Chinese academia on the whole. From this perspective, the current research literature on reclaimed water in China is mainly about technology and engineering, and less about policy and management. Therefore, the work in this paper is valuable for analyzing the utilization policies of reclaimed water in China.

## 2. Research Design

### 2.1. Research Methodology

In this paper, we used CiteSpace software for econometric analysis and atlas analysis of the literature [31,32]. CiteSpace 5.7.R2 was mainly used to draw the knowledge map, and the hot spots, frontiers, core authors, and cooperation networks in reclaimed water policy research were visualized and analyzed [33]. We used NVivo 12 Plus (QSR International: Burlington, MA, USA) for content analysis, mainly aiming at the highly cited papers, to obtain the basic value, main framework, and main content of reclaimed water utilization policy.

### 2.2. Data Sources

The data in this paper were obtained from the China National Knowledge Infrastructure and WOS databases. In the WOS system, we selected the SSCI and SCI databases, and in CNKI we selected the CSSCI and Peking University core databases. The literature search date was 15 July 2022.

In the WOS system, the search formula was ((TS = (“reclaimed water”) AND TS = (policy)) AND DT = (article), ((TS = (“reclaimed water”)) AND “((TS = (“policy”)) AND “recycled water” DT = (article)”, and a total of 196 SSCI and SCI articles were retrieved.

In the CNKI database, the search formula was “(subject = reclaimed water OR recycled water) AND (subject = policy OR governance)”; 173 CSSCI and Peking University core articles were retrieved, and the search results were screened manually. Excluding non-academic literature, such as essays, calls for papers, book reviews, news reports, meeting notices, and editorial columns, as well as repetitive and obviously irrelevant literature, 160 relevant publications were finally obtained.

## 3. Visual Analysis

### 3.1. Post Analysis

Publication analysis is a process of decomposing and analyzing the quantity, trends, and composition of published literature in a research field from different dimensions. This type of analysis can help researchers grasp the total number and annual number of publications in the research field and predict publication trends. Analyzing the publication situation, cooperative networks, and publication trends of a research field in different countries can help researchers quickly grasp the situation in terms of national attention, cooperation, national influence, and publication trends.

**Figure 1:**
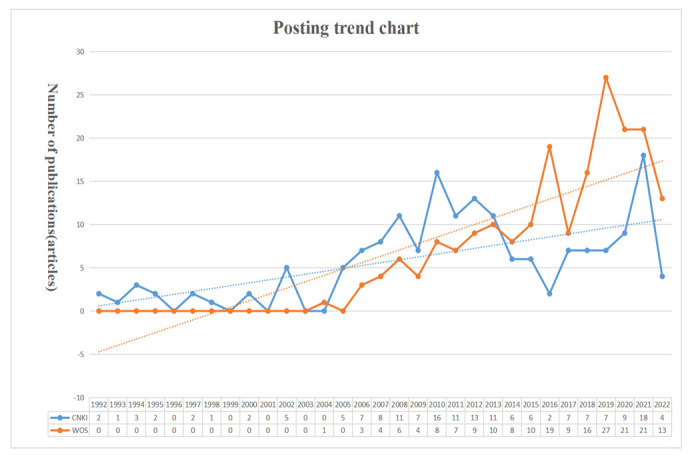
Publication trends.

Based on the number of publications in the CNKI and WOS databases, a publication trend chart was drawn (shown in Figure 1). It can be seen in Figure 1 that the domestic published literature covering reclaimed water utilization policy started in 1992. From 1992 to the retrieval date, 173 related studies were published in the CNKI database. The international published literature on reclaimed water utilization policy started in 2006. From 2006 to the retrieval date, 196 relevant studies were published in the WOS database. Considering the overall publication trend, the number of domestic and international studies on recycled water utilization policies is on the rise. The average annual number of domestic and international articles published was 5.58 and 11.53, respectively. Overall, both domestic and international research attaches great importance to reclaimed water use policies, and although international attention was late in China, it developed rapidly. International publishing after 2013 already exceeded that in China, as shown by the amount of related literature. On the whole, the research into reclaimed water utilization policy is very promising.

**Figure 2:**
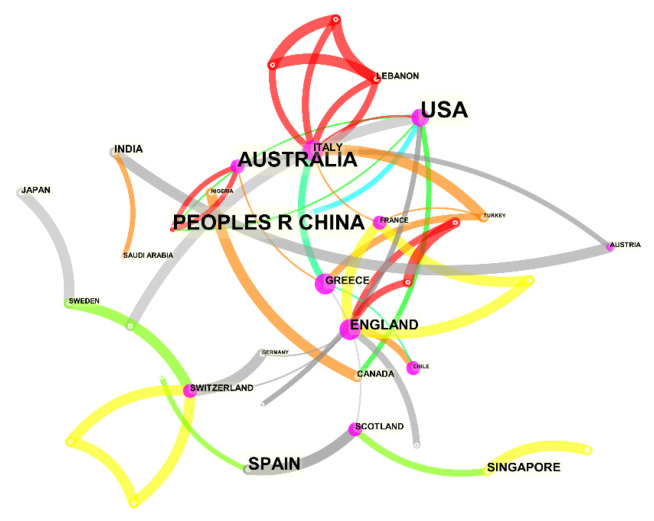
Country cooperation.

**Figure 3:**
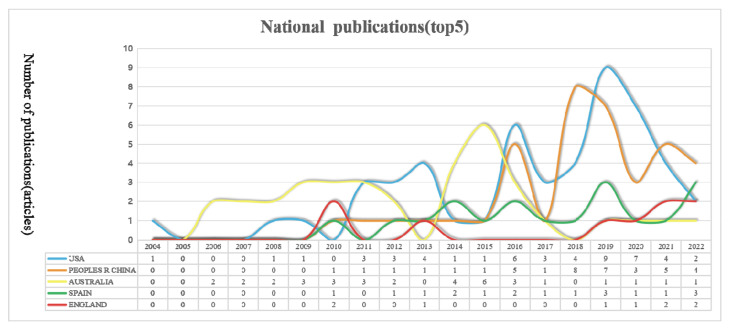
National publishing trends.

With the help of CiteSpace 5.7.R2, we analyzed the WOS data, setting the time interval as 2014–2022, the time slice as 1, and the node types as country. The selection criteria were set to G-index, k = 10, with pruning in Pathfinder, pruning sliced networks, and pruning the merged network. CiteSpace was run to obtain the cooperative publications of countries with reclaimed water utilization policies (see Figure 2 and Table 1). Then, the publication frequency data of the five countries with the most publications were imported into Excel to draw a national publication trend chart (see Figure 3).

In the WOS database, the top five countries with the highest number of articles published (see Table 1) were the USA, with 50 articles; the People’s Republic of China, with 39 articles; Australia, with 35 articles; Spain, with 18 articles; and the UK, with 9 articles. This showed that these countries all attach great importance to reclaimed water utilization policies. In particular, the United States being in the lead indicated that this country attaches the highest importance to reclaimed water utilization policies. In terms of national cooperation, the circles in Figure 2 represent hub nodes; a hub node has high centricity, and centricity refers to a node having the most short-circuit bridges to other nodes and thus having a central role in the overall network. The circle size represents centricity; the bigger the circle, the stronger the centricity. More centrality means more influence. As shown in Figure 2 and Table 1, countries with large nodes are England, Greece, Italy, the USA, and France, indicating that these are the countries with the greatest influence on the research field of reclaimed water utilization policy in the WOS database. As shown in Figure 3, in terms of national publication trends, the UK, Greece, Italy, and France all showed an upward trend, indicating that reclaimed water utilization policies will continue to attract their attention in the future.

### 3.2. Analysis of Research Subjects and Cooperative Networks

The current progress of research on reclaimed water utilization policy could not have been achieved without the unremitting efforts of certain researchers and teams. The analysis of the authors’ structural characteristics and cooperative networks reflected the core authors and their relationships in this field.

Core authors are scholars at high academic levels and with significant scientific research achievements in their field. Analyzing the core authors is helpful for understanding the research status and progress of a field. According to Price’s law, the number of core authors can be calculated as follows:

$$\mathrm{MP}=0.749\;\sqrt{NP\max}$$

MP represents the minimum number of papers published by core authors, and NPmax represents the cumulative number of papers published by the authors with the most papers in the research time interval. If the number of stable core authors accounts for 50% of the total number of papers, the field is considered to have formed a core author group.

For author visualization analysis, information on 160 items of literature retrieved from CNKI were imported into CiteSpace. We found that among scholars, HaiWen Wu had the most published literature, a total of 31 articles. According to the calculation formula, it was concluded that the value of MP was 4.17, indicating that authors with five or more publications could be regarded as core authors in the field of reclaimed water utilization policy research in China. As shown in Table 2, there are four core authors in this research field: HaiWen Wu, Lihui An, Feifei Wang, and Kun Lei, based on the number of articles published. Compared with other authors, these core authors published more articles: HaiWen Wu with 31 articles, Lihui An with 28 articles, Feifei Wang with 16 articles, and Kun Lei with 12 articles. These authors started to pay attention to reuse policy in 1992, more than 30 years ago, and had a series of achievements. Together, they published a total of 87 articles, accounting for 54.37% of the total, which was higher than the standard for core authors, indicating that a core group of domestic authors studying reclaimed water utilization policy had been formed.

In WOS, the information of 196 retrieved publications was imported into CiteSpace for a visual analysis of author status. It was found that the number of published articles related to reclaimed water utilization policy in WOS was lower than that in CNKI on the whole, and the author who published the most articles was Anna Hurlimann, with only five articles. According to the calculation formula, the value of MP was 1.67, that is, authors who published two or more articles could be regarded as core authors in the field of reclaimed water utilization policy research internationally.

There are 22 core authors in the field of reclaimed water use policy research internationally, including the 10 listed in Table 2. The others are Wenmeng Yu, Bing Zhang, David Sauri, Ali Guna, Kimberly J. Quesnel, Mengmeng Wang, Jun Wu, Dajun Shen, Jinghua Sha, Ching Leong, Mohammad Hossein Niksokhan, and Tingting Zhang, each with two articles. According to the statistics, the number of core authors is 52, and their published articles account for 26.53% of the total international papers on reclaimed water use policy, which is lower than the standard for core authors, indicating that a core group of international authors studying international reclaimed water use policy has not yet been formed.

**Figure 4:**
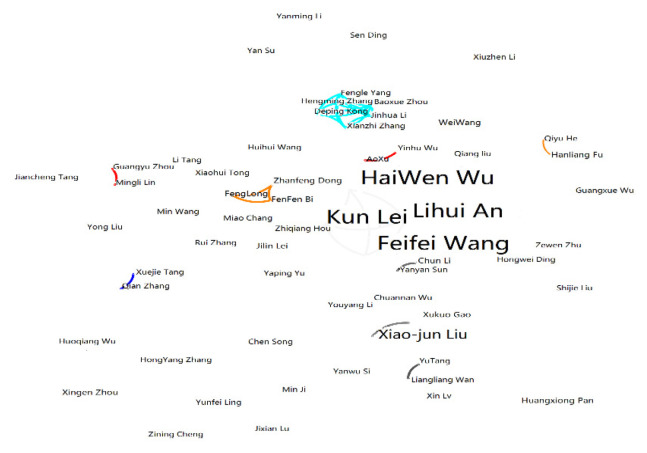
Cooperation chart of domestic authors.

**Figure 5:**
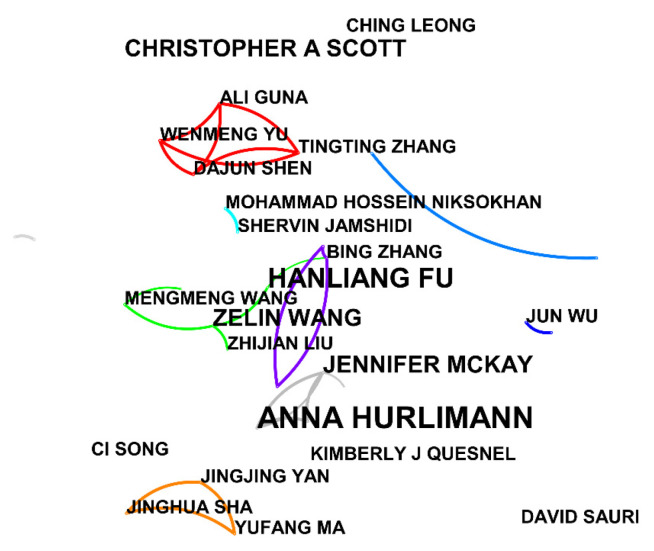
Collaboration chart of international authors.

CiteSpace was used to obtain the author cooperation network map (Figure 4 and Figure 5), in which the annual ring nodes represent authors, the node size represents centrality, and the node connection represents the existence of a cooperative relationship. It can be seen from the analysis that in CNKI (Figure 4), the number of network nodes N = 192, the number of connections E = 130, and the network density D = 0.0071, indicating that the distribution is relatively scattered. There are three research teams: the core research team composed of HaiWen Wu, Lihui An, Feifei Wang, and Kun Lei; the second team composed of FenFen Bi, Feng Long, and Zhanfeng Dong; and the third team composed of Deping Kong, Xianzhi Zhang, Baoxue Zhou, and others. On the international side (Figure 5), the number of network nodes was 185, the number of connections was 136, and the network density was 0.008, indicating that the distribution is relatively scattered. There are three research teams: one team composed of Anna Hurlimann, Jennifer Mckay, and others; the second team consisting of Ali Guna, Wenmeng Yu, Tingting Zhang, Dajun Shen, and others; and the third team consisting of Jingjing Yan, Jinghua Sha, Yufang Ma, and others. In general, there is a certain degree of cooperation among authors, but the concentration is still low and the cooperative relationships are relatively loose. Specifically, the authors in the research field of reclaimed water utilization policy show the characteristics of overall dispersion and sporadic concentration. Most of the authors are engaged in independent research, only a few have established cooperative relationships, and a cooperative network has not yet been formed.

**Figure 6:**
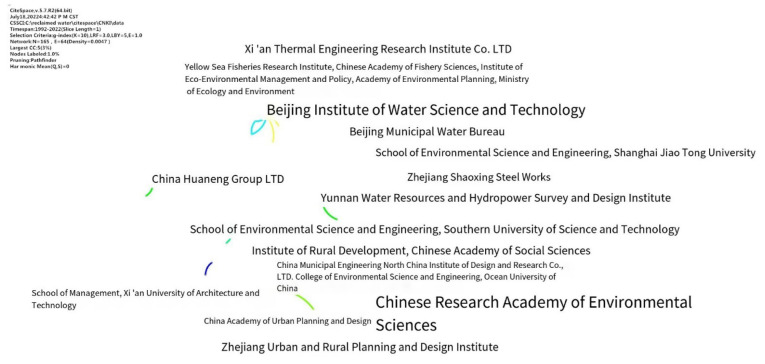
Cooperation network of domestic institutions.

**Figure 7:**
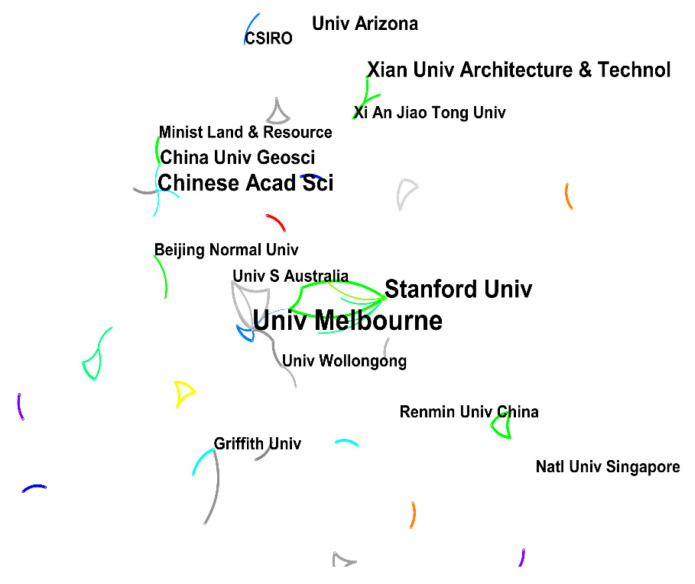
Cooperation network of international institutions.

In order to explore the cooperation of research institutions in the field of reclaimed water utilization policy, a cooccurrence analysis of the institutions mentioned in the sample literature was conducted, statistics of number of documents issued by research institutions (see Table 3) and a cooperation network map was obtained (Figure 6 and Figure 7). The size of annual rings is proportional to the number of publications, and the connection between nodes and their thickness represent cooperative relationships between institutions and their frequency. As shown in Figure 6, the number of nodes in the cooperation network of domestic research institutions was 165, the number of connections was 64, and the network density was 0.0047. In terms of the number of publications, the China Environmental Science Research Institute had the most, with 33 articles, followed by Xian Building University of Science and Technology School of Management and China Academy of Urban Planning and Design, with only 3 articles. In addition, there is a lack of cooperation between research institutions, and they conduct independent research. In the future, institutions should strengthen their cooperation, exchange theoretical ideas, and promote research development. 

As shown in Figure 7, the number of nodes in the cooperative network of international research institutions was 154, the number of lines was 97, and the network density was 0.0082. The number of publications from international institutions was less than that from domestic institutions. The University of Melbourne had the most articles with 10, followed by Stanford University with 7 and the Chinese Academy of Sciences with 6. Compared with domestic institutions, international institutions are more linked, but do not have a high level of cooperation and do not form a cooperative network. As can be seen from Figure 6 and Figure 7, there was no clear cooperation network between research institutions in either CNKI or WOS, indicating that there is no frequent or fixed relationship between research institutions.

### 3.3. Analysis of Research Hotspots

#### 3.3.1. Keyword Co-Occurrence Analysis

Keywords are words chosen by the author to summarize the topic of the article, representing the author’s highly summarized and refined academic thoughts, research themes, and research content. Therefore, keywords can also be used to analyze the research topic. At the same time, by examining the frequency of keywords, we can understand the research hotspots in the field and judge how quickly the research content is updated and the vigor of the research. The keywords in this section were automatically generated by the CiteSpace software. The principle was to select at least two words to form a keyword.

**Figure 8:**
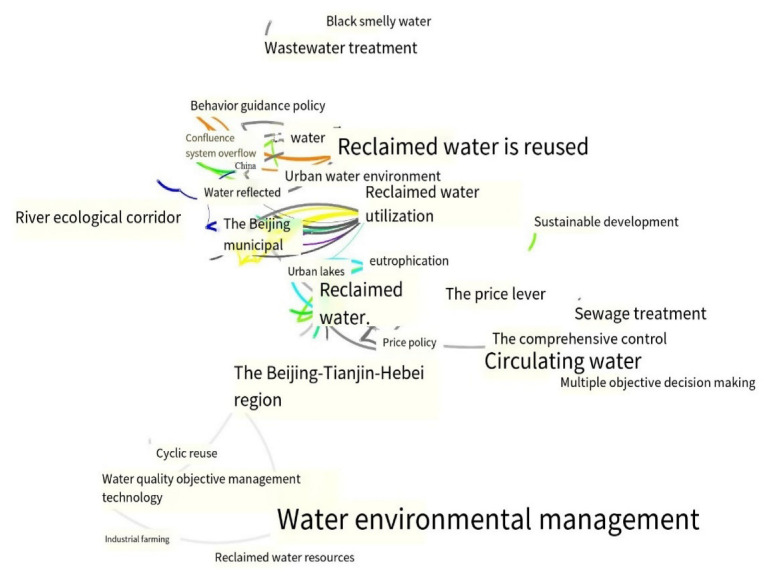
Cooccurrence of keywords in CNKI.

**Figure 9:**
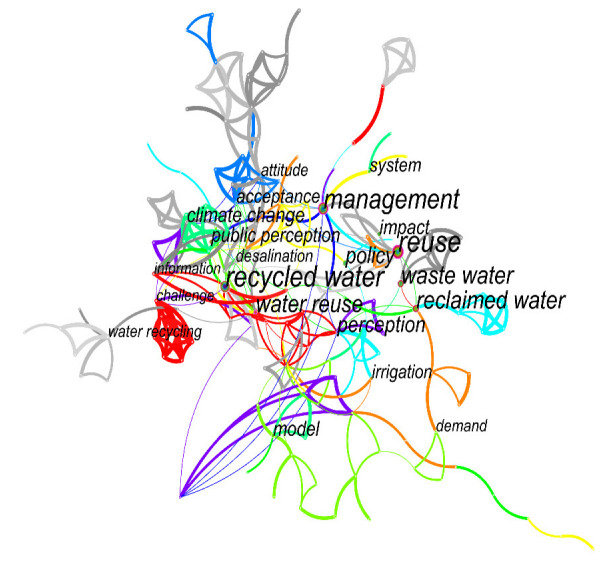
Cooccurrence of keywords in WOS.

In a knowledge graph, the research topics and hotspots of a certain field can be obtained by analyzing the keywords. In the keyword cooccurrence network constructed by CiteSpace, each node represented a keyword, and the size of the node represented frequency. Running CiteSpace, we set the node type as Keyword and the time range from 1992 to 2022 in China and 2004 to 2022 globally. We imported the research data and used the Keyword function of CiteSpace. Keyword cooccurrence maps (Figure 8 and Figure 9) and a keyword frequency and centrality scheme (see Table 4) were obtained. As can be seen from Figure 8 and Figure 9, the maps had a total of 216 domestic nodes, with 277 lines and a network density of 0.0119, and 189 international nodes, with 318 lines and a network density of 0.0179.

Keyword cooccurrence analysis is the analysis of keywords provided by authors in a dataset. According to the keywords generated by CiteSpace for domestic reclaimed water utilization policy research from 1992 to 2022 (see Table 4), it can be seen that in CNKI, except for “reclaimed water” and “recycled water”, the top 10 most frequent keywords were “Beijing–Tianjin–Hebei region”, 31 times; “reclaimed water utilization”, 16 times; “water environment management”, 11 times; “water quality objective management technology”, 11 times; “river ecological corridor”, 9 times; “reclaimed water reuse”, 6 times; “Beijing”, 6 times; and “reclaimed water plant”, 3 times. This reflects that research in this field in CNKI mainly focuses on the utilization of reclaimed water and the management of the water environment in the Beijing–Tianjin–Hebei region and Beijing. In WOS, in addition to “reclaimed water”, “recycled water”, “policy”, “water reuse”, “reuse”, “management”, “waste water”, and related keywords, the top 10 most frequent keywords included “perception”, 15 times; “climate change”, 13 times; and “impact”, 12 times. This reflects that perception, climate change, and impact are the focus of research on reclaimed water utilization policy in WOS.

#### 3.3.2. Keyword Cluster Analysis

**Figure 10:**
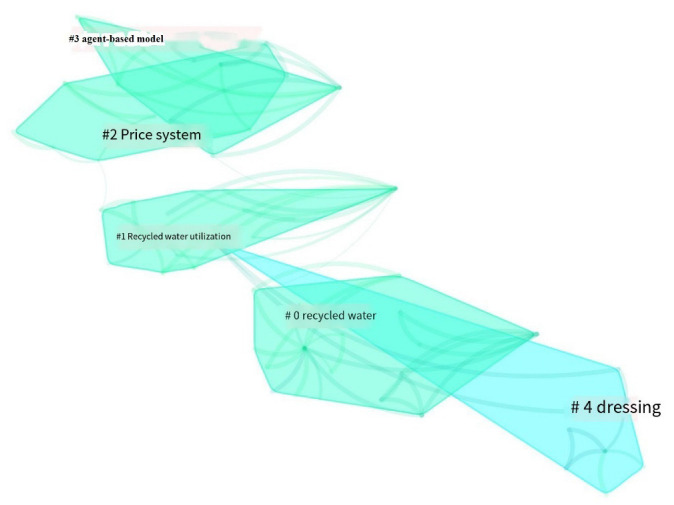
CNKI keyword clustering.

**Figure 11:**
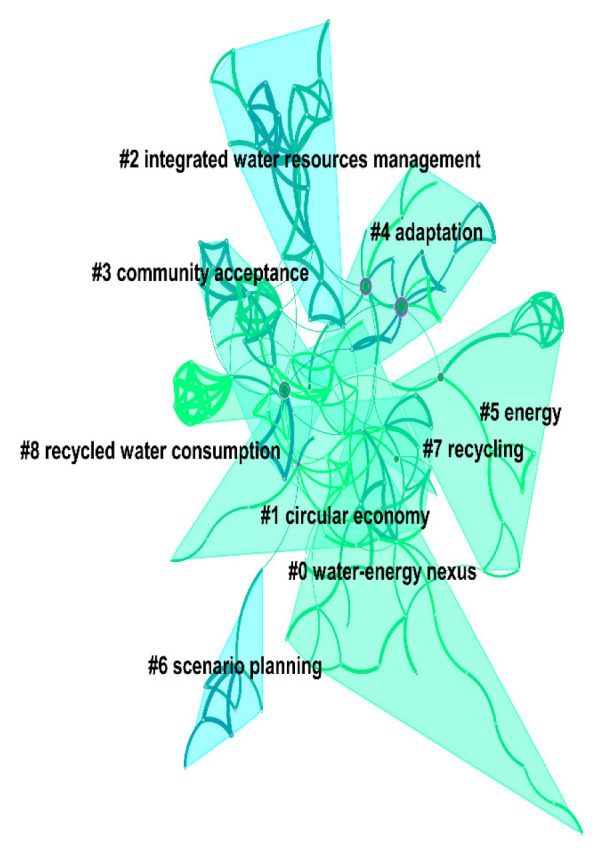
WOS keyword clustering.

According to the network structure and the clarity of clustering, CiteSpace measures the effect of mapping by two indexes: the Q value and the S value. The Q value is modularity, i.e., the module value, and its interval is [0, 1]. Q > 0.3 means that the divided community structure is significant. The S value is the weighted mean silhouette, which is the average contour value. S > 0.5 means that the clustering is reasonable, and S > 0.7 means that the clustering is efficient and convincing. As shown in Figure 10, in CNKI the Q value of the keyword cluster map was 0.9322, so its structure was significant, and the S value was 0.7359. In WOS, the Q value of the keyword cluster map was 0.748, and the S value was 0.8841, which was larger than the reasonable average contour value of 0.5 and larger than 0.7, indicating that the clustering analysis was efficient and convincing. The clustering results with significant structures and good effects were helpful for us when analyzing and grasping the overall characteristics and development trends of reclaimed water utilization policy research. As shown in Figure 10, the top five clusters in China were 0 “reclaimed water”, 1 “reclaimed water utilization”, 2 “price system”, 3 “agent-based model”, and 4 “mineral processing”. As shown in Figure 11, the top five clusters internationally were 0 “water energy”, 1 “circular economy”, 2 “integrated water resources management”, 3 “community acceptance”, and 4 “adaptation”.

The keyword cluster summary table was drawn to further explore the number of keywords contained in each cluster, the closeness of the cluster itself, the average year of keyword distribution, and the main keywords included in the cluster. As can be seen from Table 5, the mean silhouette (S value) of each cluster was greater than 0.8, indicating that each cluster was efficient and convincing. In CNKI, cluster 0 (reclaimed water) had 18 nodes in descending order; cluster 1 (reclaimed water utilization) had 14 nodes; cluster 2 (price system) had 9 nodes; cluster 3 (agent-based model) had 8 nodes; and cluster 4 (mineral processing) had 8 nodes. The average year of each cluster was between 2011 and 2012, and 2011 had the largest number, 26 clusters. From WOS, cluster 0 (water energy) had the largest number of nodes, 26; cluster 1 (circular economy) had 21 nodes; cluster 2 (integrated water resources management) had 20 nodes; cluster 3 (community acceptance) had 19 nodes; cluster 4 (adaptation) had 19 nodes; cluster 5 (energy) had 13 nodes; and clusters 6–8 (scenario planning, recycling, and recycled water consumption) had 8 nodes each. The keywords extracted according to the weighting algorithm are listed for each cluster, and the top five keywords are ranked from left to right in order of importance (only the top five are shown).

#### 3.3.3. Keyword Time Series Analysis

**Figure 12:**
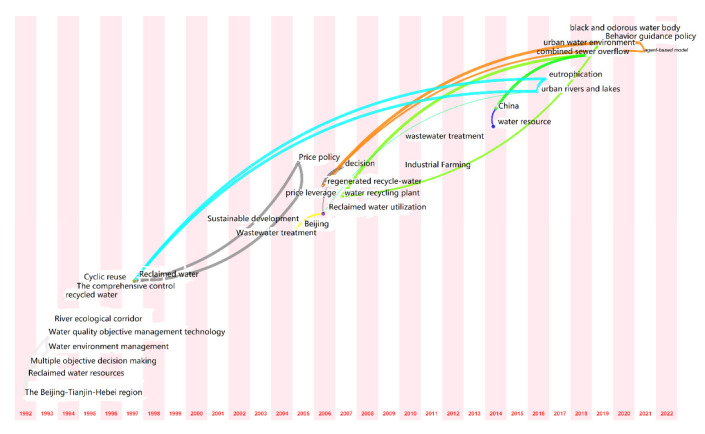
Time-zone map of research on reclaimed water utilization policies from CKNI.

**Figure 13:**
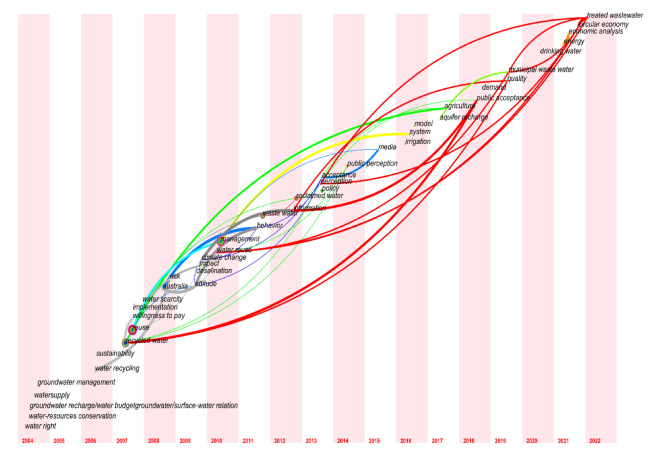
Time-zone map of research on reclaimed water utilization policies from WOS.

The research hotspots were dynamic and were not the same in each time period. CiteSpace software provided a presentation mode for a time-zone view of the literature co-citation network. Keywords were set in the time zone in which they appeared for the first time, and the time series were arranged in order from far to near; then, the keyword co-occurrence map was obtained by adjusting and embellishing. In this way, the time dimension of the research hotspots could be clearly displayed. Based on the keyword cooccurrence map, the time slice was set to 1 year, and the other settings were unchanged. We clicked “Run” to obtain the keyword cooccurrence map, then clicked “Layout” on the shortcut control board of the visualization interface and selected “Timezone View” under “Visualizations” to obtain the original map. The map was further embellished by adjusting the parameters, and the time-zone map of the hotspots of domestic research on reclaimed water utilization policies from 1992 to 2022 was obtained (Figure 12). The time-zone map of the hotspots of international research on reclaimed water utilization policies from 2004 to 2022 was also obtained (Figure 13).

As shown in Figure 12 and Figure 13, each keyword was marked according to the year in which it appeared for the first time; the more years for which it appeared, the larger the circle, and thus the larger the keywords on the scale point of the circle, which indicated the keywords that appeared most in different years. For example, in the two figures, the keyword “recycled water” first appeared in 1997 and 2007, respectively, and then reappeared several times in subsequent years, thus forming larger circles. In the figures, the lines represent connections between different keywords, and the more links between different keywords, the thicker the lines. A keyword may be associated with multiple other keywords, and there are cross-links between different keywords.

In general, reclaimed water utilization policy research has evolved from a single issue to a complex issue, gradually deepening and being improved. Over time, the relationships between research issues have become closer, and the correlation degree has increased. In contrast, domestic research on reclaimed water utilization policy started early, and over time, the research hotspots shifted from regional governance to water environmental management to water ecological governance to wastewater management to policy governance. Foreign research on reclaimed water utilization policy started late but developed rapidly. Over time, the research hotspots transitioned from water rights to water resources protection to willingness to pay to public acceptance to water quality improvement to economic analysis, indicating that international research on reclaimed water utilization policy is more specific, microscopic, and in-depth.

#### 3.3.4. Emergent Keyword Analysis

“Emergent keywords” refer to keywords that suddenly appear more frequently within a certain period, reflecting their importance in that period. Chaomei Chen defined the research frontier as a set of emergent dynamic concepts and potential research questions, which can accurately reflect the frontiers of related disciplines. Emergent keywords were used to explore the emergent dynamic concepts and potential research questions in the field of reclaimed water utilization policy, explore the reasons behind them, reflect active or cutting-edge research nodes, and help in predicting research hotspots and trends in the future. The basic principle of emergent word detection is that the frequency of the use of a keyword surges in a short period of time and the topic suddenly becomes a research hotspot, which can be considered the Baidu index of academic circles. Since the state of emergent words usually has time continuity over a period of 2 years or more, such words can be used to help predict research hotspots and trends in the future. At the same time, emergent word detection can be used to review which keywords represent hotspots in certain time periods.

**Figure 14:**
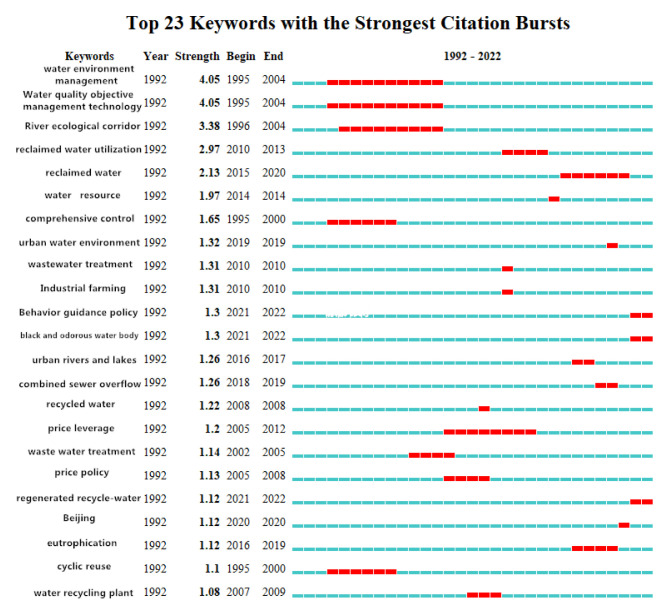
Keywork bursts in regenerated water utilization policy research from CNKI.

**Figure 15:**
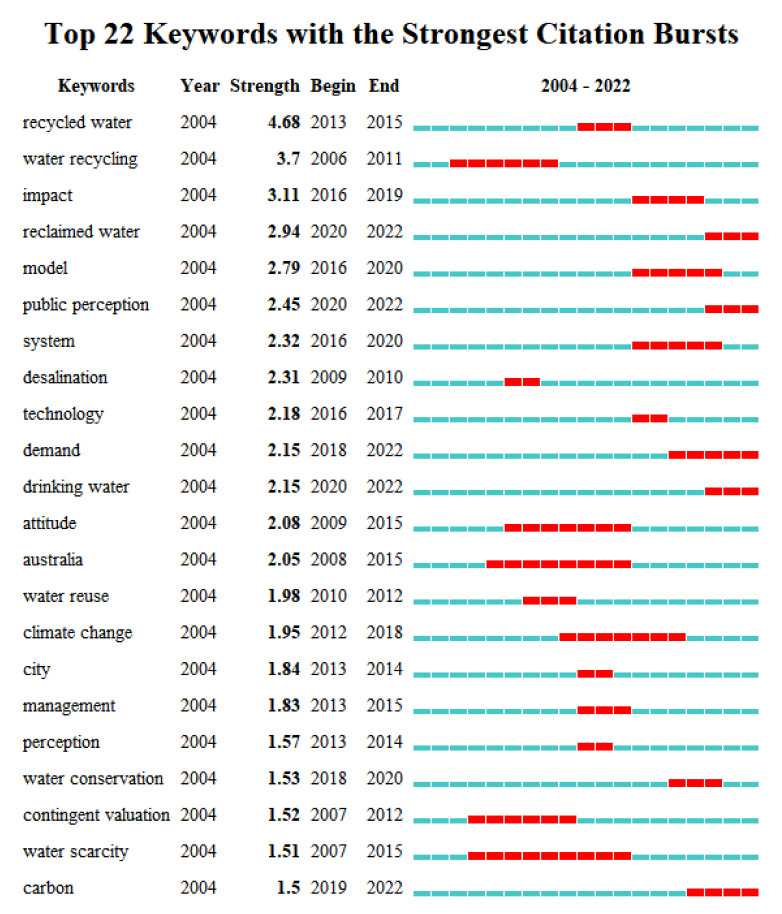
Keyword bursts in research on reclaimed water utilization policy from WOS.

When checking for keyword bursts, in CNKI, the gamma value was set to 0.3 and the parameter of minimum duration was set to 1, to obtain the graph in Figure 14. In WOS, the gamma value was set to 0.5 and the parameter of minimum duration was set to 2, to obtain the graph in Figure 15. In the map, “Begin” represents the year when the keywords emerged in the study category, “End” represents the year when the keywords disappeared, “Strength” represents the intensity of emergence, the blue block represents the unit annual time slice, and the red block represents the emergence period.

As shown in Figure 14 and Figure 15, emergent keywords from research on reclaimed water utilization policy in CNKI with higher intensity included: “water environment management” (4.05), “water quality objective management technology” (4.05), “river ecological corridor” (3.38), “water resource” (1.97), “comprehensive control” (1.65), and “urban water environment” (1.32), indicating that these were the frontier topics that domestic researchers paid more attention to in the corresponding time periods. The emergent keywords in research on reclaimed water utilization policy in WOS with high intensity were: “impact” (3.11), “model” (2.79), “public perception” (2.45), “system” (2.32), “desalination” (2.31), and “technology” (2.18), indicating that these were the frontier topics that international researchers paid more attention to in the corresponding time periods. The duration of emergent keywords in research on reclaimed water utilization policy was as follows: “water environment management” (10 years); “water quality objective management technology” (10 years); “river ecological corridor” (9 years); “price leverage” (8 years)”; and “comprehensive control” (5 years). This indicates that these keywords have been the focus of domestic scholars for a long time, and some are even hot topics. The duration of emergent keyworks in research on reclaimed water utilization policy in WOS was as follows: “water succinct” (9 years); “Australia” (8 years); “attitude” (7 years); “climate change” (7 years); and “contingent valuation” (6 years). This indicates that these keywords have been the focus of international scholars for a long time. 

According to the time component, it could be found that frontier keywords constantly changed over time, generally showing a phased evolution. Therefore, in this study, we divided the research frontiers in the field of reclaimed water utilization policy according to time stages, and we selected the keywords with high emergent intensity in each period for analysis. In China, the period 1992 to 2005 was the early stage. The frontier keywords with high emergent intensity were “water environment management” (4.05), “water quality objective management technology” (4.05), “comprehensive treatment” (1.65), “recycling” (1.1), “river ecological corridor” (3.38) and “waste water treatment” (1.14). The middle period was 2006–2014. The frontier keywords with high emergent intensity were “price leverage” (1.2), “price policy” (1.13), “reclaimed water plant” (1.08), “sewage treatment” (1.31), “industrial aquaculture” (1.31), and “water resources” (1.97). The recent period was 2015–2022. The frontier keywords with high emergent intensity were “urban rivers and lakes” (1.26), “eutrophication” (1.12), “confluence system overflow” (1.26), “urban water environment” (1.32), “Beijing City” (1.12), and “behavior guidance policy” (1.3). In WOS, 2004 to 2015 was the early stage, and the frontier keywords with high emergent intensity were “contingent valuation” (1.52), “water terrain style” (1.51), and “attitude” (2.08). The period from 2016 to 2020 was the middle stage, and the frontier keywords with high emergent intensity were “impact” (3.11), “model” (2.79), “system” (2.32), and “technology” (2.18). The recent period was 2020-2022, and the frontier keywords with high emergent intensity were “carbon”, “public perception”, and “drinking water”.

### 3.4. Analysis of Highly Cited Literature

Papers in a research field are not isolated, and they gradually form an interconnected citation network that links past and future research and shows cross-integration between disciplines through standard citations among scholars. Therefore, according to the limited citation frequency method, we ranked the 196 articles retrieved from the WOS database in descending order of citation frequency, and we retrieved papers with no fewer than 90 citations for high citation analysis. A total of 13 highly cited papers from the source literature were queried, as shown in Table 6. Similarly, the 174 articles retrieved from the CNKI database were sorted by the same method, and papers with no fewer than 30 citations were retrieved for high citation analysis, as shown in Table 7.

#### 3.4.1. Research Topic Analysis

By analyzing the research topics of the highly cited literature, we could understand the main topics of the standard literature in this research field and the main content categories of the knowledge base.

**Figure 16:**
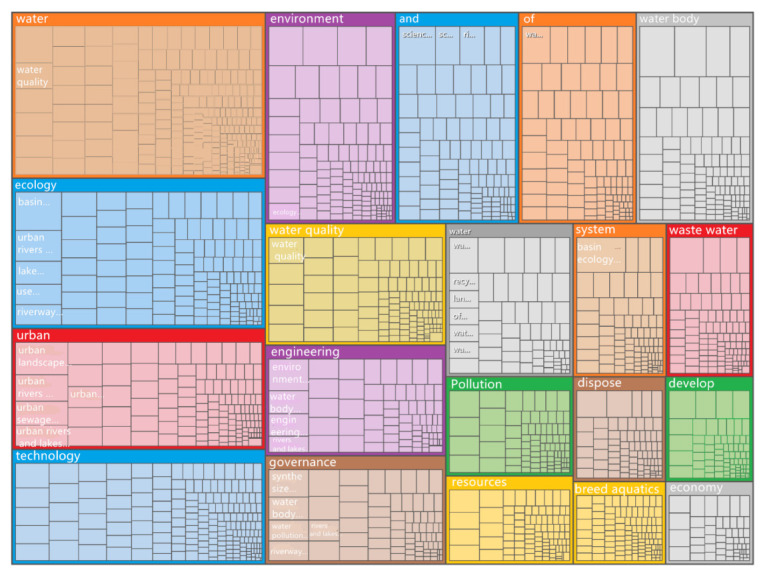
Hierarchical diagram of automatically coded topic nodes and reference points from CNKI.

The full text of 13 highly cited papers in CNKI was imported into NVivo 12 Plus, and the function “identifying topics” under the function “automatic coding” was run to obtain the topics, node numbers, and reference points of domestic research on reclaimed water utilization policies (Table 8) and a hierarchical diagram of nodes and reference points (Figure 16). As shown in Figure 16, “environment”, “ecology”, “water quality”, “system”, “sewage”, “urban”, “rivers and lakes”, “engineering”, “pollution”, “technology”, “resources”, “aquaculture”, “water body”, “waste water”, “governance”, “develop”, and “economy” were the subjects of the highly cited domestic research publications. These topics were sorted by the number of reference points, as shown in Table 7. The top 10 nodes were “watershed ecosystem”, “urban landscape water body”, “water self-purification capacity”, “urban river and lake water ecology”, “water environment quality”, “urban sewage regeneration”, “urban river and lake water body”, “comprehensive treatment”, “national environmental protection”, and “lake ecological restoration”. This shows that domestic research on reclaimed water utilization policy is mainly focused on water environment management and ecological security management.

**Figure 17:**
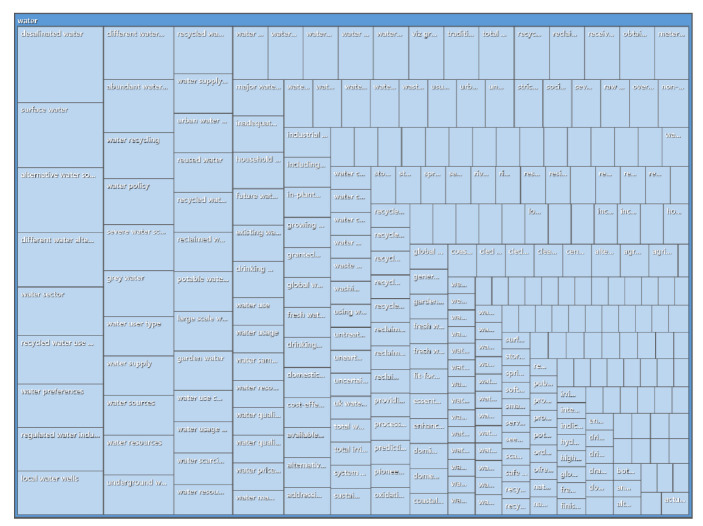
Hierarchical diagram of automatically encoded topic nodes and reference points from WOS.

The full text of 13 highly cited publications in WOS was imported into NVivo 12 Plus, and the “identifying topic” function under the “automatic coding” function was run to obtain the topic “water”. The number of nodes included in this topic and the reference points were summarized, as shown in Table 9. The hierarchical diagram of the number of main nodes and their reference points under this topic are shown in Figure 17. It can be seen in the figure that the themes identified by automatic coding were “desalinated water”, “surface water”, “abundant water”, “water recycling”, “water policy”, “reused water”, “grey water”, “water sector”, “water user type”, “recycled water use”, “water supply”, “water resources”, “underground water”, “regulated water”, and “local water wells”. Table 9 shows the automatic encoding result from WOS sorted by reference points. The top 10 selected nodes were “desalinated water”, “alternative water source”, “surface water”, “different water alternatives”, “recycled water use projects”, “water sector”, “abundant water resources”, “different water user groups”, “local water wells”, and “regulated water industry”. This shows that international research on reclaimed water utilization policy is mainly focused on the policy background, the intention of the subject and object of policy implementation, and the path of policy optimization and promotion.

#### 3.4.2. Sentiment Analysis of Research Literature

**Figure 18:**
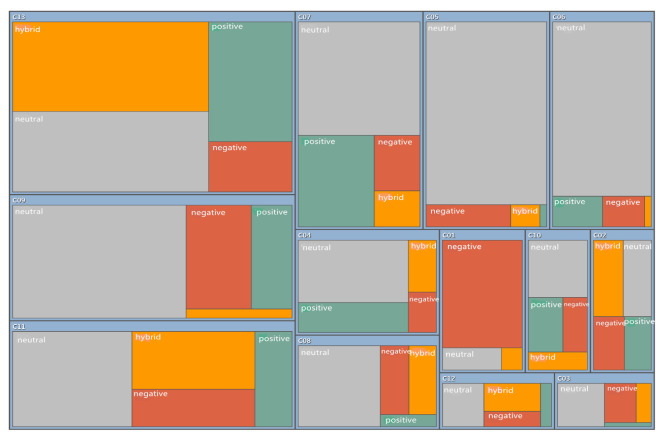
Hierarchical summary of automatically coded emotions toward highly cited literature in CNKI.

**Figure 19:**
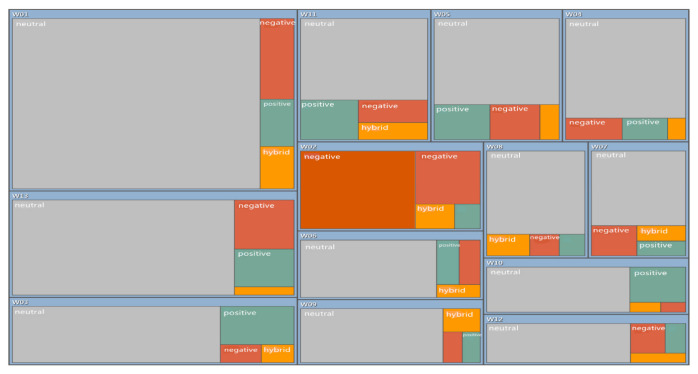
Hierarchical summary of automatically coded emotions toward highly cited literature in WOS.

Through the analysis of the highly cited literature in CNKI and WOS, and the automatic code-recognition sentiment analysis by NVivo software, a statistical table of coding points and a hierarchical chart summarizing the research literature according to sentiment were obtained. According to the statistics in Table 10, 255 nodes were coded as “very negative” in China, 135 nodes were coded as “relatively negative”, 361 nodes were coded as “relatively positive”, and 10 nodes were coded as “very positive”. It can be seen from Figure 18 that the majority of nodes were coded as “neutral” and “hybrid”, so the results of the analysis of scholars in China at present show that attitudes toward the policy of using reclaimed water are still complex, but also that the existing policies have made certain achievements, and there are still substantial development prospects. According to the statistics in Table 11, for the international research, 230 nodes were coded as “very negative”, 601 nodes were coded as “relatively negative”, 595 nodes were coded as “relatively positive”, and 205 nodes were coded as “very positive”. It can be seen in Figure 19 that almost half of the research was coded as “neutral”, and the proportion of positive and negative attitudes tended to be balanced. The dominant attitude of international researchers is that international policy has certain applicability, but there are obvious problems, so it remains to be further adjusted and perfected.

## 4. Conclusions

With the advantages of high performance, recyclability, and an “in-place” effect, reclaimed water resources have become the best choice to solve the contradiction between supply and demand. The visualization of research in CNKI and WOS demonstrated the following: (1) The number of articles on reclaimed water policy at home and abroad showed an upward trend, researchers attach great importance to the subject, and the research prospects are broad. (2) The UK, Greece, Italy, the United States, and France have great influence in the field of reclaimed water utilization policy research. The international influence of China’s research needs to be improved. (3) There is a lack of communication and cooperation among researchers in the field of reclaimed water utilization policy, and a cooperative network with close and benign interactions has not yet been formed. (4) The hotspots of reclaimed water utilization policy research in China are mainly focused on regional governance, and there is not enough emphasis on policy and management, while foreign countries pay more attention to policy and management. (5) From the perspective of research trends, behavior guidance policy and black and smelly water will become the hotspots of domestic reclaimed water utilization policy research, while public perception, demand, drinking water, and carbon will become the hotspots of international research. (6) The highly cited papers in the domestic research on reclaimed water utilization policies focus on water environment management and ecological security management, whereas the highly cited papers in the international research focus on the background of reclaimed water utilization policy, policy implementation and object intention, and the optimization of the ascension path toward stronger policies. (7) Domestic and foreign researchers have a predominately rational and emotional attitude toward reclaimed water use policy, indicating that the current policy has certain applicability, but there are also problems, such as a lack of outstanding policy orientation and the operability of promotion measures, which need to be further adjusted and improved.

## 5. Suggestions for Optimizing Reclaimed Water Utilization Policies in China

To sum up, China is still in the initial development stage of reclaimed water use policies and must strengthen the theoretical research. Research on reclaimed water use should pay attention to technological innovation, and it needs not only the government to step up efforts to promote it, but also people from all walks of life to raise awareness and form a force to promote domestic and international theoretical research and technical cooperation. Realizing coordinated promotion among the government, enterprises, and society would be conducive to the development of reclaimed water utilization not only in China, but also globally.

Based on the above research conclusions, this paper puts forward the following optimization suggestions for China’s reclaimed water utilization policies.

### 5.1. Strengthen the Top-Level Design of Reclaimed Water Utilization

At present, the central level of reclaimed water utilization is directly related only to the laws and regulations of ecological environment staff, the general office of the National Development and Reform Commission, the general office of housing and urban–rural development, and the general office of the Ministry of Water Resources with regard to printing and distributing notifications of regional renewable water recycle pilot implementation plans (ring for water no. 28 (2021)). This indicates that the top-level design of reclaimed water utilization in China is seriously deficient and is still in the pilot stage of “crossing the river by feeling the stones”, which is not conducive to overall planning and systematic guidance and needs to be strengthened urgently. Therefore, in addition to strengthening the top-level design, the specific content, such as development goals, phased tasks, planning and design standards, safety supervision, and performance assessment, should be clarified at the national level, so as to improve the utilization of reclaimed water from the management level to the highest level.

### 5.2. Improve the Reclaimed Water Utilization Policy System

At present, China’s renewable water utilization policies are insufficient, mainly in terms of resources and environmental protection, urban construction regulations, pricing policy supplementations, and the adoption of fiscal and tax policies, and no synergy has been formed. In other words, the current policies in China are mainly regulatory, and there is a serious shortage of incentive policy tools, mainly based on government advocacy and guidance, and a benign market operation mechanism has not been established. The policy system needs to be improved urgently. Therefore, we should constantly improve the policy system and introduce a series of preferential policies for reclaimed water utilization to stimulate the reclaimed water supply. In terms of taxation, loans, land use, and other aspects, more practical preferential policies should be applied to enterprises and individuals investing in reclaimed water. In addition, we should increase financial subsidies for the main users of reclaimed water to stimulate consumption and use. We should give full play to the regulatory role of price leveraging, keep the prices of reclaimed water and tap water within a reasonable range, and guide more people to choose reclaimed water when it is safe to do so.

### 5.3. Strengthen the Regulation of Reclaimed Water Utilization Policies

To establish a safety supervision system for reclaimed water utilization with multi-party participation, in addition to relying on to the regulatory function of the government, it is also necessary to actively encourage third-party organizations and the public to participate. On the one hand, we should emphasize the scientific nature of reclaimed water use in regulatory and professional terms, along with design production, distribution, use, and other aspects involved in all aspects of water quality, complete with a strong, comprehensive law enforcement team to safeguard the work put in place. On the other hand, we should incorporate the supervisory power of third-party evaluation institutions and the public, strengthen publicity and education, and guide people to actively participate and play a role in supervising the utilization of reclaimed water. In addition, it is necessary to actively promote the development of technology for reclaimed water utilization, strengthen the construction of intelligent monitoring systems, give full play to the important role of technical monitoring, and improve the information level.

## Figures and Tables

**Table 1:** Statistics of national cooperative publishing (Top 10).

Serial Number	Count (Top 10)			Centrality (Top 10)		
	Country	Count	Year	Country	Centrality	Year
1	USA	50	2004	England	0.58	2010
2	People’s R China	39	2010	Greece	0.54	2007
3	Australia	35	2006	Italy	0.53	2017
4	Spain	18	2010	USA	0.38	2004
5	England	9	2010	France	0.31	2020
6	Singapore	7	2015	Switzerland	0.29	2010
7	Greece	6	2007	Scotland	0.29	2007
8	Italy	6	2017	Australia	0.25	2006
9	India	6	2010	Chile	0.25	2017
10	Switzerland	4	2010	Austria	0.15	2010

**Table 2:** Comparison of number of articles published by research authors (top 10).

Serial Number	CNKI			WOS		
	Author	Count	Year	Author	Count	Year
1	HaiWen Wu	31	1992	Anna Hurlimann	5	2007
2	Lihui An	28	1992	Hanliang Fu	4	2018
3	Feifei Wang	16	1992	Jennifer Mckay	3	2007
4	Kun Lei	12	1992	Zelin Wang	3	2018
5	Xiao-jun Liu	3	2005	Christopher A Scott	3	2011
6	FenFen Bi	2	2021	Shervin Jamshidi	2	2016
7	Jilin Lei	2	2010	Jingjing Yan	2	2021
8	HanLiang Fu	2	2021	Ci Song	2	2016
9	Huangxiong Pan	2	1993	Yufang Ma	2	2021
10	Feng Long	2	2021	Zhijian Liu	2	2018

**Table 3:** Comparison of number of publications by research institutions (top 10).

Institution	Count	Year	Institution	Count	Year
Chinese Research Academy of Environmental Sciences	33	1992	University of Melbourne	10	2007
School of Management, Xi’an University of Architecture and Technology	3	2005	Stanford University	7	2017
China Academy of Urban Planning and Design	3	2020	Chinese Academy of Science	6	2011
Shaoxing Steel Works in Zhejiang Province	2	1993	Xian University of Architecture and Technology	5	2017
Beijing Water Resources Planning and Design Research Institute	2	2010	University of Arizona	4	2011
Institute of Rural Development, Chinese Academy of Social Sciences	2	2018	China University of Geoscience	4	2016
Business School, Hohai University	2	2008	CSIRO	3	2010
Institute of Eco-Environmental Management and Policy, Academy of Environmental Planning, Ministry of Ecology and Environment	2	2021	Renmin University of China	3	2012
Yellow Sea Fisheries Research Institute, Chinese Academy of Fishery Sciences	2	2010	Xi An Jiao Tong University	3	2018
China Municipal Engineering North China Design and Research Institute Co., Ltd.	2	2020	National University of Singapore	3	2015

**Table 4:** Keyword frequency comparison.

Keywords	Word Frequency	Centricity	Year	Keywords	Count	Centrality	Year
Beijing–Tianjin–Hebei region	31	0	1992	Reuse	45	0.28	2007
Reclaimed water	16	0.05	1997	Recycled water	42	0.12	2007
Reclaimed water utilization	12	0.07	2006	Management	35	0.27	2010
Water environmental management	11	0	1993	Waste water	24	0.1	2011
Water quality objective management techniques	11	0	1993	Reclaimed water	23	0.2	2012
River ecological corridor	9	0	1993	Water reuse	22	0.27	2010
Reuse reclaimed water	6	0.02	2006	Policy	17	0.03	2013
Beijing municipal	6	0.04	2005	Perception	15	0.18	2013
Circulating water	5	0	1994	Climate change	13	0.12	2009
Recycled water	3	0.01	2007	Impact	12	0	2009

**Table 5:** Clustering summary and comparison.

Database	Label	Number of Nodes	Outline of Value	Year	Keywords
CNKI	0	18	0.975	2011	Reclaimed water (2.83, 0.1); Price leverage (2.83, 0.1); Water resource fee (2.83, 0.1); Reclaimed water utilization (2.83, 0.1); Temporary storage (1.4, 0.5).
	1	14	0.968	2012	Reclaimed water utilization (4.71, 0.05); Ministry of Education (2.32, 0.5); Treatment and reuse (2.32, 0.5); Changjiang Scholars Program (2.32, 0.5); Organic toxicants (2.32, 0.5).
	2	9	1	2012	Price system (6.54, 0.05); Water resources (6.54, 0.05); Government management (6.54, 0.05); Reclaimed water (0.19, 1.0); Price leverage (0.19, 1.0)
	3	8	0.975	2011	Agent-based model (5.36, 0.05); Public acceptance (5.36, 0.05); Behavior guidance policy (5.36, 0.05); BP neural network (5.36, 0.05); Reuse of reclaimed water (5.36, 0.05).
	4	8	1	2000	Mineral processing (5.86, 0.05); Comprehensive treatment (5.86, 0.05); Lead–zinc ore (5.86, 0.05); Wastewater (5.86, 0.05); Reclaimed water (0.25, 1.0); Price leverage (0.25, 1.0).
WOS	0	23	0.901	2017	Water–energy nexus (8.78, 0.005); Water supply (8.62, 0.005); Beijing (8.62, 0.005); Megacity (4.28, 0.05); Reclaimed water reuse (4.28, 0.05).
	1	21	0.779	2016	Circular economy (7.17, 0.01); Treated SBR (7.17, 0.01); Social capital (3.56, 0.1); Vineyards (3.56, 0.1); Other water supply mix (3.56, 0.1).
	2	20	0.897	2009	Integrated water resources management (5.27, 0.05); Community attitudes (5.27, 0.05); Organic contaminant (5.27, 0.05); Advanced water treatment (5.27, 0.05); Conjoint analysis (5.27, 0.05).
	3	20	0.79	2013	Community acceptance (7.51, 0.01); Climate change (7.51, 0.01); Greenhouse gas emissions (3.73, 0.1); Cultural theory (3.73, 0.1); Toowoomba (3.73, 0.1).
	4	19	0.925	2012	Adaptation (4.05, 0.05); Another treatment ecosystem (4.05, 0.05); Emergy-based indicator (4.05, 0.05); Australian water recycling (4.05, 0.05); Alternative supplies (4.05, 0.05).
	5	13	0.909	2017	Energy (8.32, 0.005); Economic analysis (8.32, 0.005); Sefidrud (8.32, 0.005); Seasonal demand (4.12, 0.05); Carbon (4.12, 0.05).
	6	8	0.952	2007	Scenario planning (6.25, 0.05); Ecologically sustainable development (6.25, 0.05); Sustainable urban development (6.25, 0.05); Mega-drought (6.25, 0.05); WaterSim 5 (6.25, 0.05).
	7	8	0.887	2018	Process (10.41, 0.005); Public perception (5.15, 0.05); Safety (5.15, 0.05); Urban communities (5.15, 0.05); Environmental protection (5.15, 0.05).
	8	8	0.951	2019	Recycled water consumption (7.35, 0.01); Recycled water utilization potential (7.35, 0.01); Price advantage (7.35, 0.01); Lock-in effect (7.35, 0.01); Under-supply (7.35, 0.01).

**Table 6:** Highly cited articles in WOS from 2004 to 2022.

Serial Number	Article Title	Authors	Citations (Times)
1	Gender attitude towards environmental protection: a comparative survey during COVID-19 lockdown situation [34]	Dhenge, Ghadge, Ahire, Gorantiwar, Shinde	273
2	Antibiotics threats on vegetables and the perils of low income nations practices [35]	Inyinbor, Tsopmo, Udenigwe	153
3	Assessment of agricultural land suitability for irrigation with reclaimed water using geospatial multi-criteria decision analysis [36]	Paul, Negahban-Azar, Shirmohammadi, Montas	126
4	An Overview of Managed Aquifer Recharge in Mexico and Its Legal Framework [37]	Cruz-Ayala, Megdal	118
5	Assessing the public perceptions of treated wastewater reuse: opportunities and implications for urban communities in developing countries [38]	Akpan, Omole, Bassey	114
6	Local recycled water in Sydney: A policy and regulatory tug-of-war [39]	Watson, Mukheibir, Mitchell	104
7	Revealing the economic value of managed aquifer recharge: Evidence from a contingent valuation study in Italy [40]	Damigos, Tentes, Balzarini, Furlanis, Vianello	102
8	Reusing wastewater to cope with water scarcity: Economic, social and environmental considerations for decision-making [41]	Garcia, Pargament	100
9	Farmers’ Attitudes towards Irrigating Crops with Reclaimed Water in the Framework of a Circular Economy [42]	Lopez-Serrano, Velasco-Munoz, Aznar-Sanchez, Roman-Sanchez	96
10	Simultaneous synthesis of property-based water reuse/recycle and interception networks for batch processes [43]	Ng, Foo, Rabie, EI-Halwagi	91
11	Determinants of the acceptance of domestic use of recycled water by use type [44]	Moya-Fernandez, Lopez-Ruiz, Guardiola, Gonzalez-Gomez	91
12	Interplay of Message Frame and Reference Point on Recycled Water Acceptance in Green Community: Evidence from an Eye-Tracking Experiment [45]	Fu, Xue, Wu, Zhu, Niu, Lai, Hou	91
13	Developing Novel Approaches to Tracking Domestic Water Demand Under Uncertainty-A Reflection on the Up Scaling of Social Science Approaches in the United Kingdom [46]	Browne, Medd, Anderson	90

**Table 7:** Highly cited articles in CNKI from 1992 to 2022.

Serial Number	Article Title	Authors	Citation (Times)
1	China’s water resources status and prospects for future development [47]	Wang, Wang, Yang, Xi, Shi, Dong, Zhang, Zhou	596
2	Technical analysis of urban black and smelly water treatment and long-term improvement and maintenance of water quality [48]	Hu, Sun, Xi, Zhao	185
3	Status quo and countermeasures of urban sewage recycling in China [49]	Ye, Liu	164
4	Strategic thinking on the structure of mariculture industry in China [50]	Lei	142
5	Analysis of treatment technology and measures for urban black and smelly water [51]	Liu, Xu, Song, Song, Sun, Zhao	87
6	Study on the impact of water resources policy on water resources carrying capacity in Beijing [52]	Fan, Liu, Guo, Wang, Jiang	81
7	Feasibility analysis of the application of condensation reheat composite technology to wet plume control in coal-fired power plants [53]	Shu, Yang, Ye, Wei, Wang, Wang	65
8	Cause analysis and treatment of water pollution in Beijing Park [54]	Wang, Li	54
9	Empirical analysis of urban sewage recycling in Japan [55]	Zhang, Liu, Yang	49
10	Analysis on the prospects of the development of recycling aquaculture model in China [56]	Luo, Zhu	41
11	Problems faced by urban water environmental governance and long-term governance model [57]	Hu, Sun, Chen, Wu, Li, Zhong	38
12	Research on municipal sewage reclamation and water resources recycling [58]	Liu, Wu	33
13	Case analysis and problem diagnosis of reclaimed water recharge type river and lake water quality improvement project in Beijing [59]	Zhang, Liu, Sun, Qi	31

**Table 8:** Summary of topic nodes and reference points of CNKI automatic coding.

Serial Number	Node	Reference Points	Serial Number	Node	Reference Points
1	Watershed ecosystems	126	11	Water treatment engineering	69
2	Urban landscape water body	123	12	Water quality	66
3	Water body self-purification ability	110	13	Bypass treatment process	63
4	Urban river and lake water ecology	101	14	Water pollution treatment	62
5	Water environmental quality	93	15	Water environment management	61
6	Municipal sewage regeneration	90	16	River ecological function	61
7	Urban rivers and lakes	84	17	River ecological management	61
8	Comprehensive control	81	18	Technical measures	60
9	National environmental protection	78	19	Water development research	59
10	Lake ecological restoration	75	20	Ecological security	56

**Table 9:** Summary of automatically coded topic nodes and reference points from WOS.

Serial Number	Node	Reference Points	Serial Number	Node	Reference Points
1	Desalinated water	14	11	Water preferences	8
2	Alternative water source	12	12	Grey water	7
3	Surface water	12	13	Severe water scarcity	7
4	Different water alternatives	10	14	Water policy	7
5	Recycled water use projects	9	15	Water recycling	7
6	Water sector	9	16	Recycled water projects	6
7	Abundant water resources	8	17	Underground water sources	6
8	Different water user groups	8	18	Water resources	6
9	Local water wells	8	19	Water sources	6
10	Regulated water industry	8	20	Water supply	6

**Table 10:** Summary of results of sentimental analysis of highly cited literature in CNKI.

Serial Number	Article Title	Very Negative	More Negative	More Positive	Very Positive
C01	Water resources status and future development in China	6	3	5	1
C02	Technical analysis of urban black and smelly water treatment and water quality long-term improvement and maintenance	26	11	37	0
C03	Current situation and countermeasures of urban sewage recycling in China	12	7	10	0
C04	Strategic thinking on the structure of mariculture industry in China	19	12	61	4
C05	Analysis of treatment techniques and measures for urban black and smelly water bodies	12	4	10	1
C06	Impact of water resources policy on water resources carrying capacity in Beijing	5	7	11	0
C07	Feasibility analysis of the application of condensation reheat composite technology to wet plume control in coal-fired power plants	11	6	31	1
C08	Analysis of causes and treatment status of water pollution in Beijing parks	29	8	22	1
C09	An empirical analysis of urban sewage recycling in Japan	15	14	18	0
C10	Analysis of prospects for the development of recycling aquaculture model in China	14	15	34	0
C11	Problems facing urban water environmental governance and long-term governance model	61	24	49	2
C12	Research on municipal sewage reclamation and water resources recycling	20	17	31	0
C13	Case analysis and problem diagnosis of reclaimed water recharge type river and lake water quality improvement project in Beijing	25	7	42	0
Combined		255	135	361	10

**Table 11:** Summary of results of sentiment analysis of highly cited literature in WOS.

Serial Number	Article Title	Very Negative	More Negative	More Positive	Very Positive
W01	Gender attitude towards environmental protection: a comparative survey during COVID-19 lockdown situation	14	58	60	25
W02	Antibiotics threats on vegetables and the perils of low income nations practices	45	91	36	14
W03	Assessment of agricultural land suitability for irrigation with reclaimed water using geospatial multi-criteria decision analysis	10	23	66	35
W04	An Overview of Managed Aquifer Recharge in Mexico and Its Legal Framework	16	35	31	8
W05	Assessing the public perceptions of treated wastewater reuse: opportunities and implications for urban communities in developing countries	20	65	68	20
W06	Local recycled water in Sydney: A policy and regulatory tug-of-war	16	28	40	14
W07	Revealing the economic value of managed aquifer recharge: Evidence from a contingent valuation study in Italy	25	45	32	12
W08	Reusing wastewater to cope with water scarcity: Economic, social and environmental considerations for decision-making	15	50	49	10
W09	Farmers’ Attitudes towards Irrigating Crops with Reclaimed Water in the Framework of a Circular Economy	15	50	49	10
W10	Simultaneous synthesis of property-based water reuse/recycle and interception networks for batch processes	3	15	28	10
W11	Determinants of the acceptance of domestic use of recycled water by use type	25	56	65	14
W12	Interplay of Message Frame and Reference Point on Recycled Water Acceptance in Green Community: Evidence from an Eye-Tracking Experiment	14	53	31	17
W13	Developing Novel Approaches to Tracking Domestic Water Demand Under Uncertainty: A Reflection on the Up Scaling of Social Science Approaches in the United Kingdom	12	32	40	16
Combined		230	601	595	205

## Data Availability

The data used to support the findings of this study are available from the corresponding author upon request.
